# Ginsenoside Rh2 Improves Cardiac Fibrosis via PPARδ–STAT3 Signaling in Type 1-Like Diabetic Rats

**DOI:** 10.3390/ijms18071364

**Published:** 2017-06-26

**Authors:** Shih-Hsiang Lo, Chao-Tien Hsu, Ho-Shan Niu, Chiang-Shan Niu, Juei-Tang Cheng, Zhih-Cherng Chen

**Affiliations:** 1 Division of Cardiology, Department of Internal Medicine, Zhongxing Branch of Taipei City Hospital, Taipei 10341, Taiwan; loshsean@gmail.com; 2 Department of Nursing, Tzu Chi University of Science and Technology, Hualien 97041, Taiwan; nhs580113@yahoo.com.tw (H.-S.N.); ncs@ems.tcust.edu.tw (C.-S.N.); 3 Department of Pathology, E-DA Hospital, I-Shou University, Yanchao, Kaohsiung 82401, Taiwan; ed103797@edah.org.tw; 4 Department of Cardiology and Department of Medical Research, Chi-Mei Medical Center, Yong Kang, Tainan 71003, Taiwan; 5 Institute of Medical Sciences, Chang Jung Christian University, Guiren, Tainan 71101, Taiwan; 6 Department of Pharmacy, Chia Nan University of Pharmacy & Science, Jean-Tae 71701, Taiwan

**Keywords:** ginsenoside Rh2, PPARδ, STAT3, cardiac fibrosis, type-1 diabetes, STZ rats

## Abstract

Ginsenoside Rh2 (Rh2) is an active principal ingredient contained in ginseng (*Panax ginseng* Meyer), a medicinal herb used to enhance health worldwide. The present study is designed to investigate the effect of Rh2 on myocardial fibrosis in diabetic rats. In a streptozotocin-induced model of type-1 diabetic rats (STZ-diabetic rats), the increased fasting blood glucose levels and heart weight/body weight (HW/BW) ratio were substantially alleviated by Rh2. Moreover, Rh2 improved cardiac performance in STZ-diabetic rats. Histological results from Masson staining showed that Rh2 attenuated cardiac fibrosis in STZ-diabetic rats. The effects of Rh2 were reversed by GSK0660 at a dose sufficient to inhibit peroxisome proliferator-activated receptor δ (PPARδ) in STZ-diabetic rats. The role of PPARδ was subsequently investigated in vitro. Rh2 restored the decreased PPARδ expression level in high glucose-cultured cardiomyocytes. Moreover, increased protein levels of fibrotic signals, including signal transducer and activator of transcription 3 (STAT3), connective tissue growth factor (CCN2) and fibronectin, were reduced by Rh2 in high glucose-cultured cardiomyocytes. These effects of Rh2 were reversed by GSK0660 or siRNA specific for PPARδ Taken together, PPARδ activation may inhibit STAT3 activation to reduce CCN2 and fibronectin expression in diabetic rats with cardiac fibrosis. Moreover, Rh2 improves cardiac function and fibrosis by increasing PPARδ signaling. Therefore, Rh2 is suitable to develop as an alternative remedy for cardiac fibrosis.

## 1. Introduction

Cardiovascular complications have been described as an important cause of death in patients with diabetes [1]. Diabetic patients easily develop heart failure [2]. Diabetic cardiomyopathy (DCM) is mainly identified with myocardial dysfunction without other heart diseases [3]. The prevalence of diabetes continuously increases, and its complications threaten human health. Therefore, the development of novel therapeutics, including phytotherapy, is extremely important.

Hyperglycemia and oxidative stress are associated with DCM [4]. Hyperglycemia-derived free radicals play an important role in DCM [5]. Hyperglycemia, a central condition in the pathogenesis of diabetes, induces the activation of the signal transducer and activator of transcription 3 (STAT3) signaling pathway, which is integral to various types of tissue damage, including myocardial dysfunctions [6]. Connective tissue growth factor (CCN2) expression is increased with pathologic fibrosis [7]. Moreover, CCN2 is introduced to mediate the occurrence and development of DCM [8]. Fibronectin is another extracellular matrix, and the fibronectin extra domain-A (ED-A) is crucial in myofibrosis [9]. STAT3 has been documented to promote the transcription of target genes, including CCN2 [10] and other genes [11], via direct interactions with consensus sites in the promoter regions. Recently, drug-induced peroxisome proliferator-activated receptor δ (PPARδ) activation has been shown to attenuate STAT3 expression for the improvement of DCM in rats [12]. Therefore, the PPARδ signaling pathway appears important in DCM.

Ginseng (*Panax ginseng* Meyer) is an herbal food that has been widely applied for many years and exhibits cardiovascular benefits through its main active ingredient, ginsenoside [13]. The contained ginsenosides may bestow on ginseng various pharmacological actions [14]. The ginsenosides Rb and Rg1 produce cardioprotective effects [15]. Ginsenoside Rh2, the active ingredient extracted from red ginseng and with the molecular formula C_36_H_62_O_8_, has been demonstrated to improve ischemic brain injury in rats [16]. Moreover, Rh2 exhibited anticancer activity in many cancer cells [17] with low toxicity to normal cells [18]. Recently, Rh2 has been shown to protect doxorubicin-induced cardiac damage [19]. Rh2 may also attenuate hyperglycemia in diabetic rats [20]. Moreover, ginseng has been documented to improve heart function via PPARδ in diabetic rats [21]. Recently, the activation of PPARδ has been demonstrated to attenuate STAT3 expression [22]. Therefore, it would be interesting to screen the effect of Rh2 on myocardial fibrosis in diabetes and investigate whether the effects are dependent on the PPARδ signaling pathway.

In the present study, we investigated the effects of Rh2 on cardiac fibrosis induced by hyperglycemia in both rats and cultured cardiomyocytes. Furthermore, we characterized the association of PPARδ with an Rh2-induced decrease of fibrotic signals, the CCN2 and fibronectin expression levels, in the diabetic heart.

## 2. Results

### 2.1. Changes in Blood Glucose Levels and Cardiac Fibrotic Parameters in Rats

During a 28-day period, the blood glucose levels in the Rh2-treated STZ rats were gradually decreased (Day 0: 351.0 ± 13.2; Day 14: 315.5 ± 16.4; Day 28: 237.4 ± 15.9). The body weights and heart weights of the rats in the STZ-diabetic group were substantially lower than those in the normal control group, whereas the blood glucose level was significantly increased in the STZ-diabetic group. However, the increased cardiac weight indices and hyperglycemia were both substantially alleviated by Rh2 at an effective dose [19] in the STZ-diabetic group (Table 1). The rats continuously treated with Rh2 showed a recovery of cardiac function compared with the vehicle (+dp/dtmax, 2301.1 ± 53.1 vs. 2089.4 ± 58.4 mmHg/s; −dp/dtmax, 1246.7 ± 35.5 vs. 931.3 ± 51.3 mmHg/s). Furthermore, these effects of Rh2 in the STZ rats were inhibited by GSK0660 at a dose sufficient to block PPARδ as previously described [23]. However, these effects of Rh2 were not present in normal rats.

### 2.2. Changes in Heart Tissues

**Figure 1:**
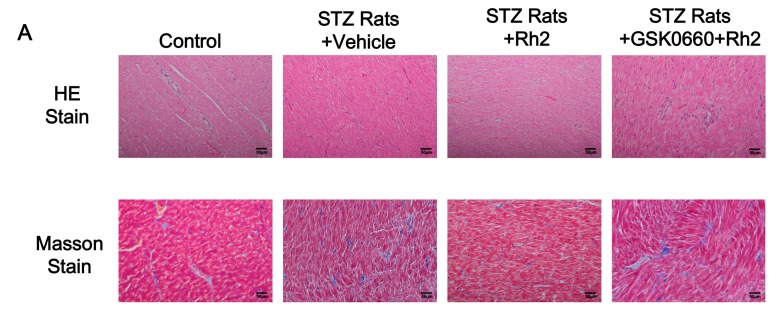

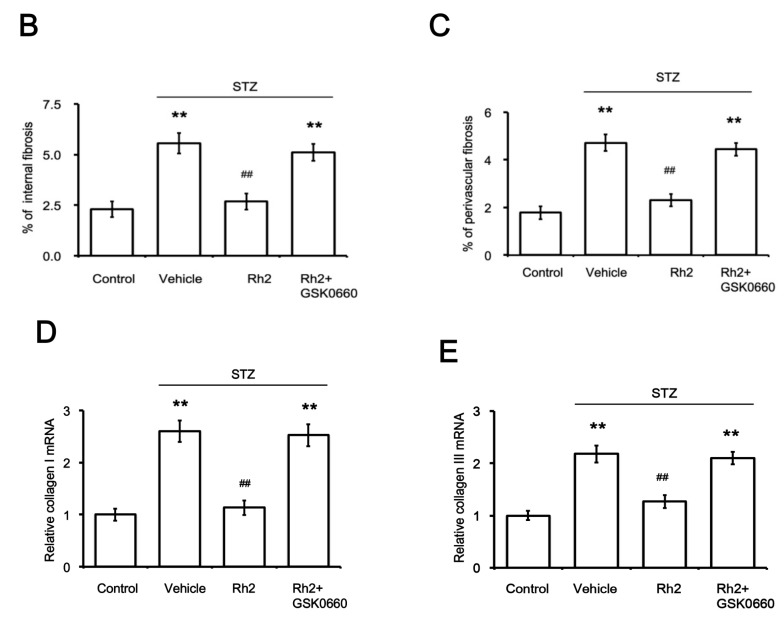
Effects of ginsenoside Rh2 on heart histology. Photomicrographs of the myocardium in the normal control group (Control), the streptozotocin-induced type-1 diabetic (STZ-diabetic) group (STZ Rats + Vehicle), the Rh2-treated STZ-diabetic group (STZ Rats + Rh2), and the GSK0660-pretreated and Rh2-treated STZ-diabetic group (STZ Rats + GSK0660 + Rh2) were characterized by staining with hematoxylin–eosin (HE Stain) and Masson’s trichrome (Masson Stain): (**A**) histological section of myocardium, the scale bar is 50 μM; (**B**) quantitative analysis of the internal fibrosis; (**C**) quantitative analysis of the perivascular fibrosis; (**D**) mRNA levels of collagen I; and (**E**) mRNA levels of collagen III. The results are presented as the mean ± SEM (*n* = 8 per group). ** *p* < 0.01 vs. control group; ^#^^#^ *p* < 0.01 vs. diabetic group (two-way ANOVA with a post hoc Tukey test).

To investigate the role of Rh2 in myocardial fibrosis, the myocardial collagen content was analyzed. Masson’s trichrome staining of heart sections demonstrated greater fibrosis in the interstitial and perivascular regions of the myocardium in the diabetes group than in the control group. In the normal heart tissue, a small extracellular matrix and some fibroblasts were observed. Diabetes significantly increased the collagen deposition in the rat hearts (Figure 1A). Moreover, the mRNA levels of type I and type III collagen were substantially up regulated in the diabetic rats (Figure 1B–E). In the Rh2 treated group, the collagen deposition and the gene expression of type I and type III collagen were substantially reduced in the rat myocardial tissues. However, the effects of Rh2 were reversed by GSK0660 at an effective dose to block PPARδ [23] and the noticeable expression of interstitial collagen fibers was revealed. A marked fibrotic response was obtained again.

### 2.3. Effect of Rh2 on Cardiac Performance in Anesthetized Diabetic Rats

The cardiac performance (+dP/dt_max_ and −dP/dt_max_) in the normal rats indicated responses consistent to a previous report [24]. Furthermore, the cardiac performance significantly decreased in the diabetic rats compared with the normal rats, and Rh2 treatment restored the dP/dt_max_ in the diabetic rats (Table 1). However, this action of Rh2 was blocked by GSK0660 at a dose sufficient to inhibit PPARδ as previously described [23].

### 2.4. Effects of Rh2 on Expression Levels of PPAR and Fibrotic Signals in Streptozotocin-Induced Type-1 Diabetic Rat (STZ-Diabetic Rat) Hearts

**Figure 2:**
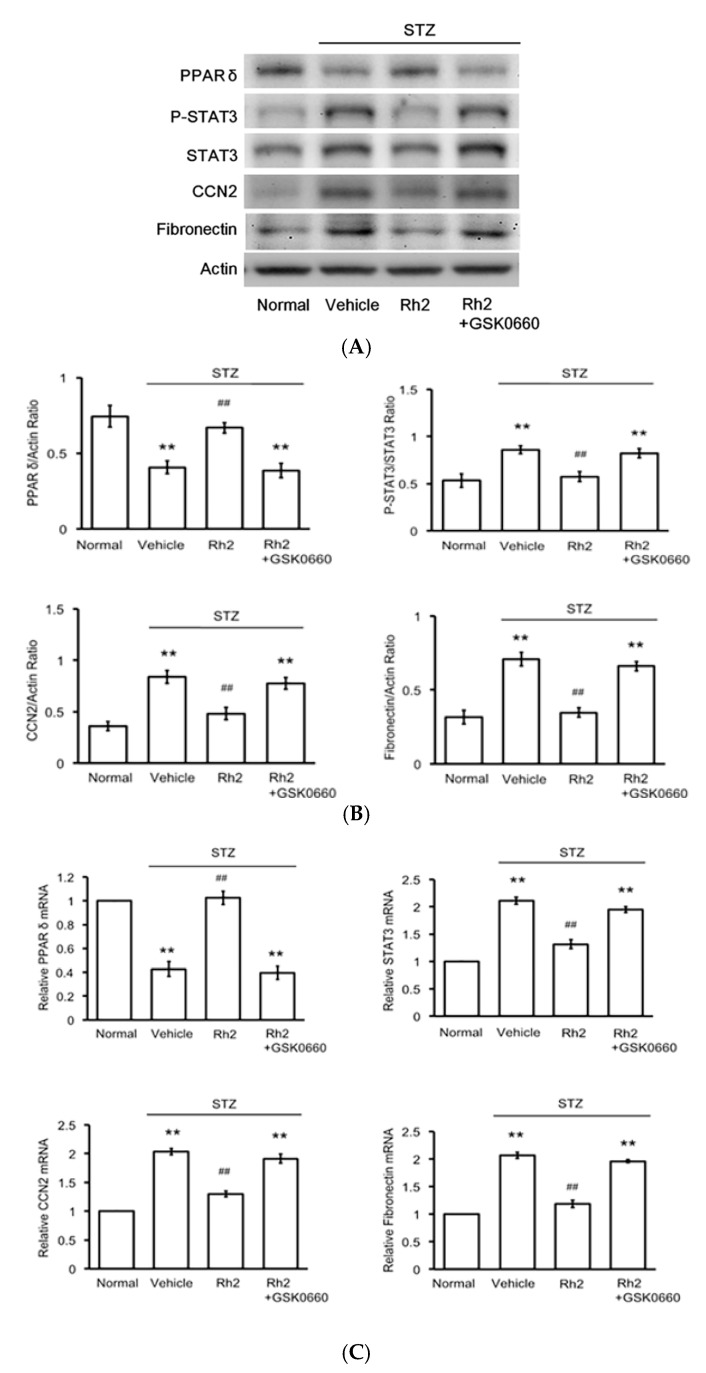
Effects of ginsenoside Rh2 on the expression levels of STAT3, connective tissue growth factor (CCN2) and fibronectin activated by peroxisome proliferator-activated receptor δ (PPARδ) in rat hearts. STZ-induced diabetic rats (STZ) were treated with or without ginsenoside Rh2 for 28 days. The rats were subsequently sacrificed, and the expression levels of PPARδ phosphorylated signal transducer and activator of transcription 3 (p-STAT3), STAT3, CCN2, and fibronectin were measured using Western blot analysis: Representative blots are presented (**A**); and the quantification of each signal relative to the internal control was performed for comparison (**B**); the mRNA levels of the previously described signals were also measured using real-time PCR (**C**). The results are presented as the mean ± SEM (*n* = 6 per group). ** *p* < 0.01 compared with the control rats and ^##^ *p* < 0.01 compared with the vehicle treated diabetic group (two-way ANOVA with a post oc Tukey test).

In contrast to the reduction in the PPARδ levels, the protein levels of p-STAT3, STAT3, CCN2 and fibronectin were substantially higher in the hearts of the STZ-induced diabetic rats than the normal control levels. However, Rh2 treatment elevated the PPARδ levels and attenuated the p-STAT3, STAT3, CCN2 and fibronectin levels in the hearts of the STZ-induced diabetic rats (Figure 2A,B). Moreover, the decreased STAT3, CCN2 and fibronectin expression in the Rh2-treated diabetic rats was reversed in the hearts of the GSK0660-pretreated group (Figure 2C). The change in PPARδ expression by Rh2 was also blocked by GSK0660 in the same manner.

### 2.5. Changes in Expression Levels of Peroxisome Proliferator-Activated Receptor (PPAR) and Fibrotic Signals by Hyperglycemia Are Reversed by Rh2 in H9c2 Cardiomyocytes

**Figure 3:**
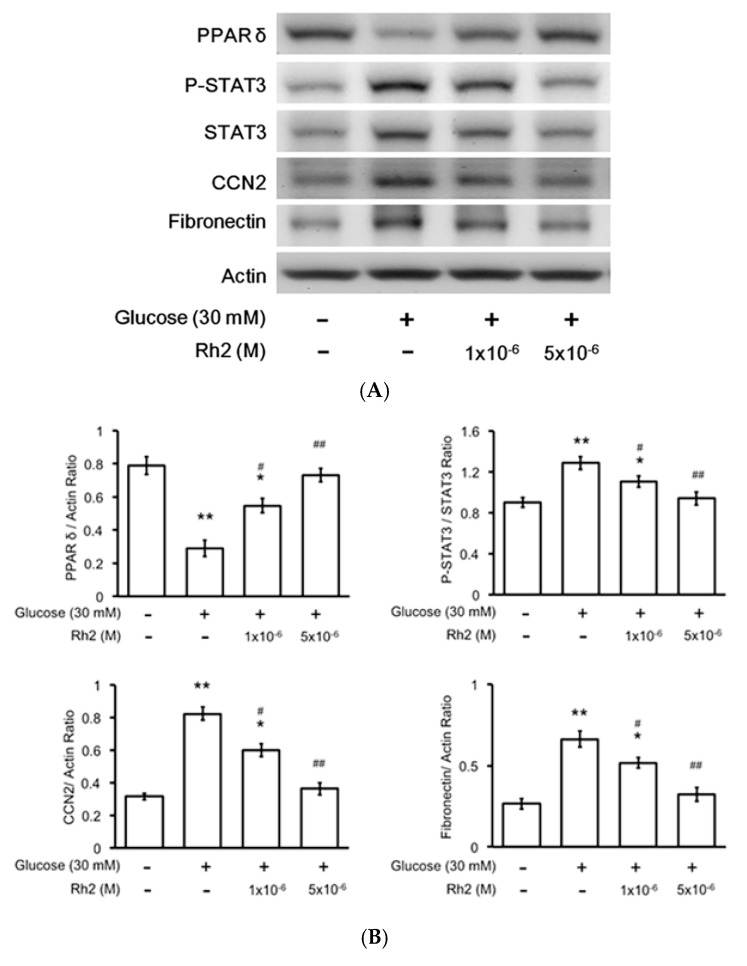

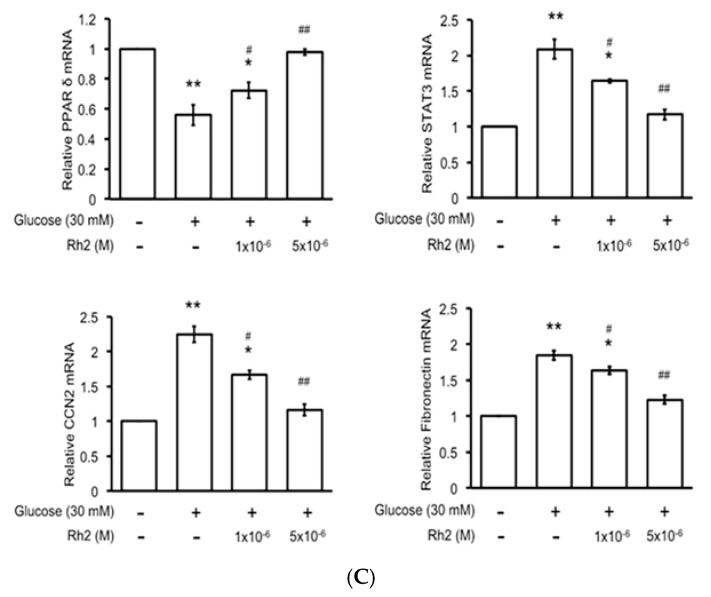
Effects of ginsenoside Rh2 on the expression levels of PPARδ STAT3, CCN2 and fibronectin in cultured H9c2 cells treated with high glucose media. We investigated the changes in PPARδ phosphorylated STAT3 (p-STAT3), STAT3, CCN2, and fibronectin protein levels induced by Rh2 at two doses in cultured cardiomyocytes after incubation with high glucose (30 mM) media for 24 h. The expression levels in normal medium (5.5 mM glucose) without treatment were used as the control. Representative blots are presented (**A**); and the quantification of each signal relative to the internal control was performed for comparison (**B**); the mRNA levels of the previously described signals were also measured using real-time PCR (**C**); the results are presented as the mean ± SEM (*n* = 6 per group). * *p* < 0.05 and ** *p* < 0.01 compared with the control (first column) and ^#^ *p* < 0.05 and ^##^ *p* < 0.01 compared with the vehicle-treated cells under high glucose media (second column) (two-way ANOVA with a post hoc Tukey test).

**Figure 4:**
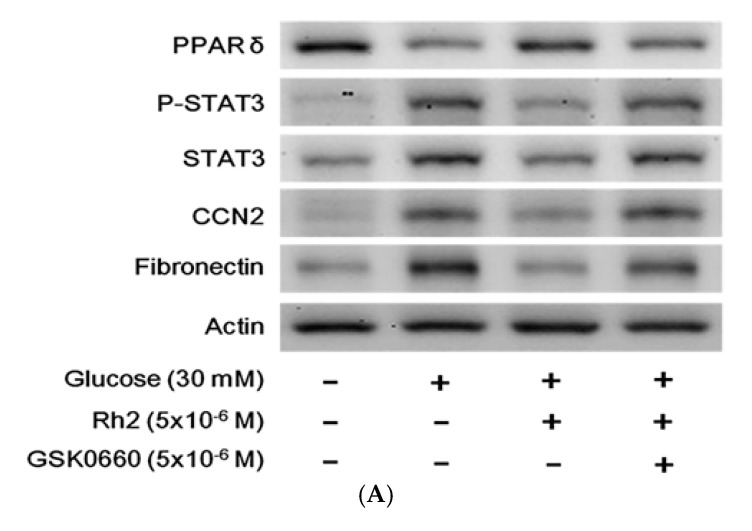

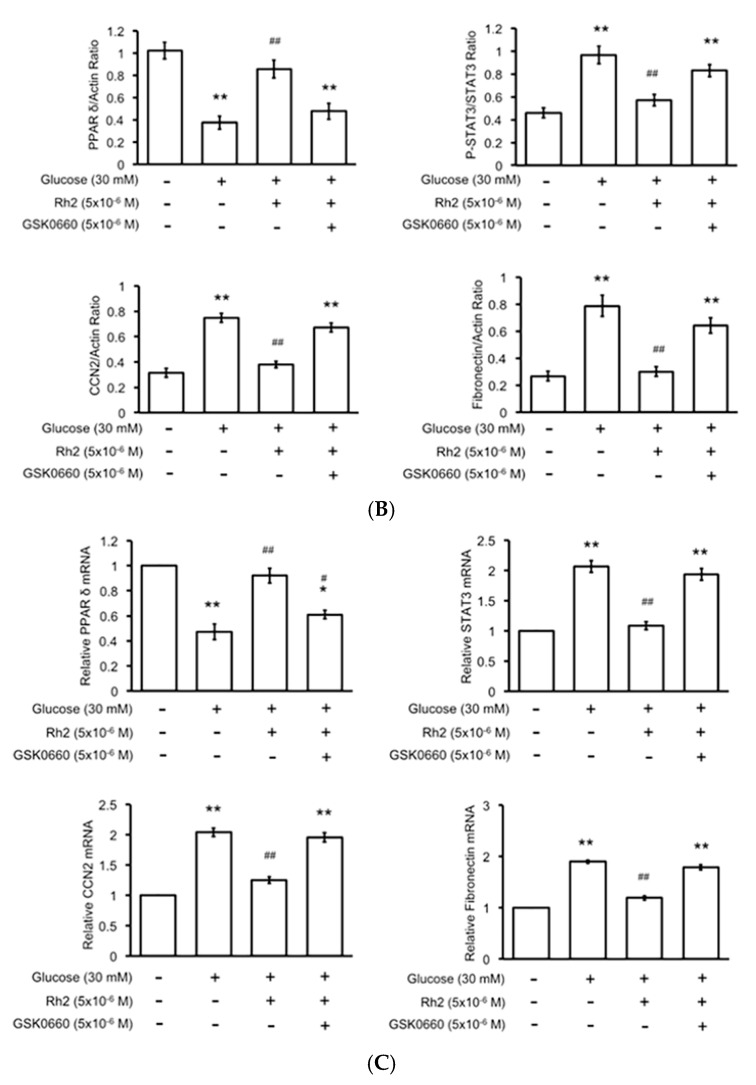
Effect of PPARδ blockade on the effects of ginsenoside Rh2 in cultured H9c2 cells under hyperglycemic conditions. Cultured H9c2 cells were pretreated with GSK0660, followed by 24-h treatment with Rh2. The H9c2 cells cultured in normal medium without treatment were used as a control. The cardiomyocytes were subsequently harvested, and the expression levels of PPARδ phosphorylated STAT3 (p-STAT3), STAT3, CCN2, and fibronectin were measured. Representative blots are presented (**A**); and the quantification of each signal relative to the internal control was performed for comparison (**B**); the mRNA levels of the previously described signals were also measured using real-time PCR (**C**). The results are presented as the mean ± SEM (*n* = 6 per group).* *p* < 0.05, ** *p* < 0.01 compared with the control (first column) and ^#^ *p* < 0.05, ^##^ *p* < 0.01 compared with the vehicle-treated cells under high glucose media (second column) (two-way ANOVA with a post hoc Tukey test).

We subsequently used the cultured cardiac H9c2 cell line to mimic the changes in diabetic hearts. The effects of hyperglycemia on the expression levels of PPARδ and fibrotic signals were identified in the cultured cardiac cell line exposed to 30 mM glucose for 24 h, as previously described [23]. The p-STAT3/STAT3, CCN2 and fibronectin expression levels increased in these cardiomyocytes, whereas a reduction of the PPARδ levels was identified under high glucose conditions (Figure 3A,B). Therefore, high glucose increases the STAT3 activation (p-STAT3/STAT3) and CCN2 or fibronectin expression levels in addition to the reduction of the PPARδ levels in cultured H9c2 cells that were the same as those observed in the hearts of the diabetic rat. Moreover, the changes in the mRNA levels of PPARδ STAT3, CCN2 and fibronectin were similarly identified in the high glucose-treated cells (Figure 3C). Rh2 treatment increased the expression of both the protein and mRNA levels of PPARδ. Rh2 also effectively attenuated the increased expression of p-STAT3/STAT3, CCN2 and fibronectin. However, these effects of Rh2 (Figure 4A) were reversed by pretreatment with GSK0660 (Figure 4B,C) at an effective dose to inhibit PPARδ [23].

### 2.6. Effects of Rh2 on Fibrosis-Related Gene Expression Were Impaired by siRNA Specific for PPAR in Cardiomyocytes

**Figure 5:**
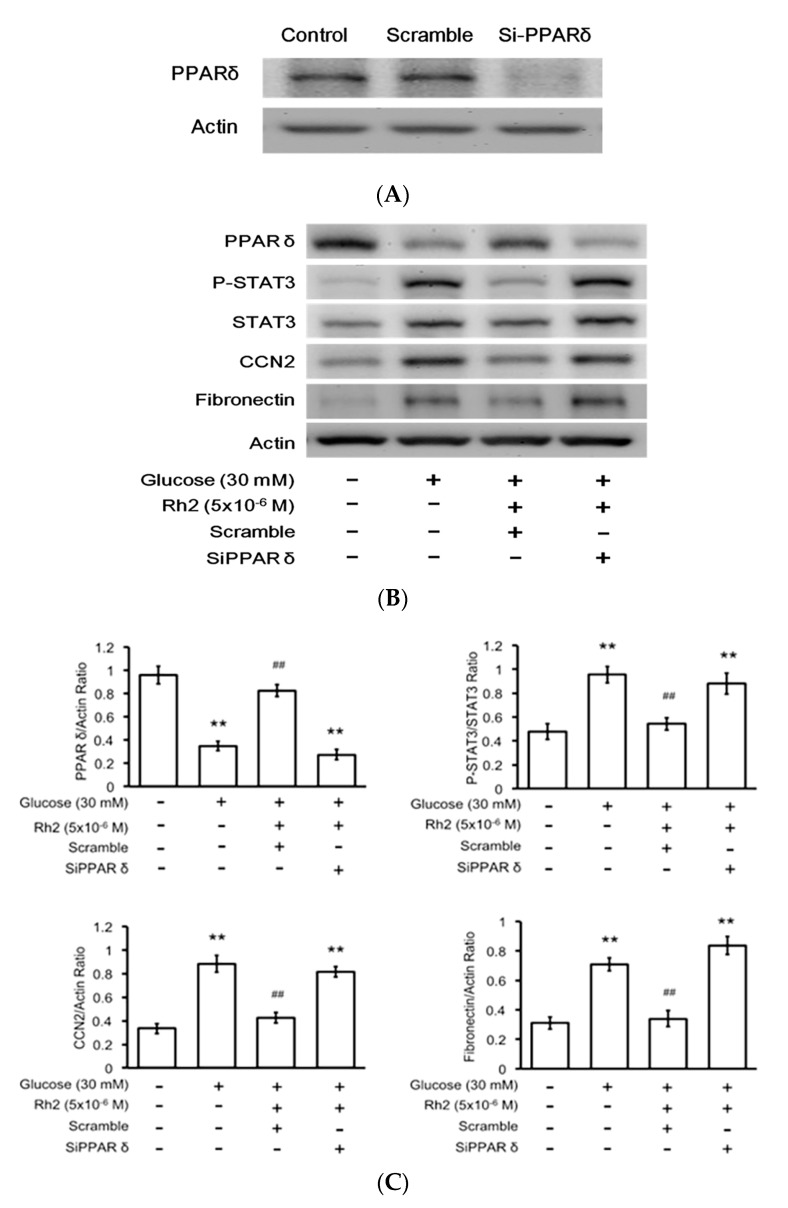

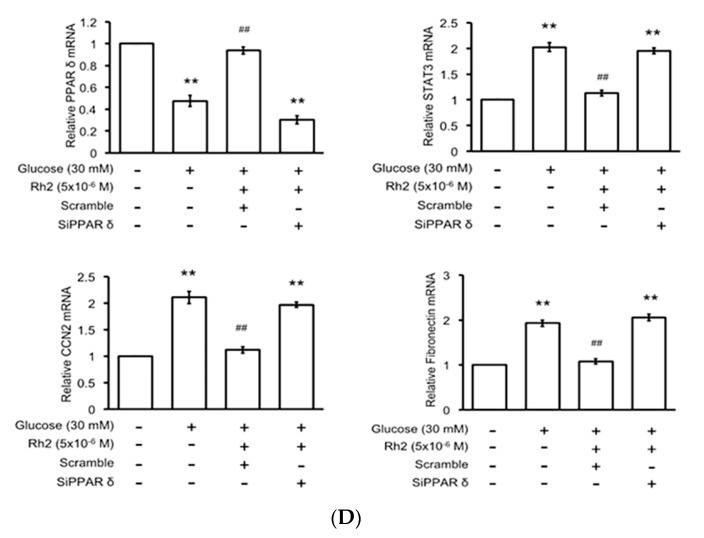
Effect of PPARδ silencing on the effects of ginsenoside Rh2 in cultured H9c2 cells under hyperglycemic conditions. Cultured H9c2 cells were transfected with siRNA specific for PPARδ or scrambled controls prior to 24-h treatment with Rh2. H9c2 cells cultured in normal medium without treatment were used as a control. Successful knockout of PPARδ in H9c2 cells was confirmed by immunoblotting (**A**). The expression levels of PPARδ phosphorylated STAT3 (p-STAT3), STAT3, CCN2, and fibronectin were measured. Representative blots are presented (**B**), and the quantification of each signal relative to the internal control was performed for comparison (**C**); the mRNA levels of the previously described signals were also measured using real-time PCR (**D**). The results are presented as the mean ± SEM (*n* = 6 per group). ** *p* < 0.01 compared with the control (first column) and ^##^ *p* < 0.01 compared with the vehicle-treated cells under high glucose media (second column) (two-way ANOVA with a post hoc Tukey test).

The transfection efficiency of the siRNA specific for the *PPAR*δ gene was confirmed via Western blot analysis (Figure 5A). The transfected H9c2 cells showed a substantially lower expression of PPARδ (Figure 5B). Moreover, Rh2-induced changes in the fibrosis-related gene expression levels were identified in the cells transfected with the scrambled control; however, all changes disappeared in the siRNA-treated cells (Figure 5C). The mRNA levels of these fibrosis-related signals modified by Rh2 were expressed in the same manner (Figure 5D).

### 2.7. Effects of Rh2 on Oxidative Damage Were Impaired by siRNA Specific for PPAR in Cardiomyocytes

**Figure 6:**
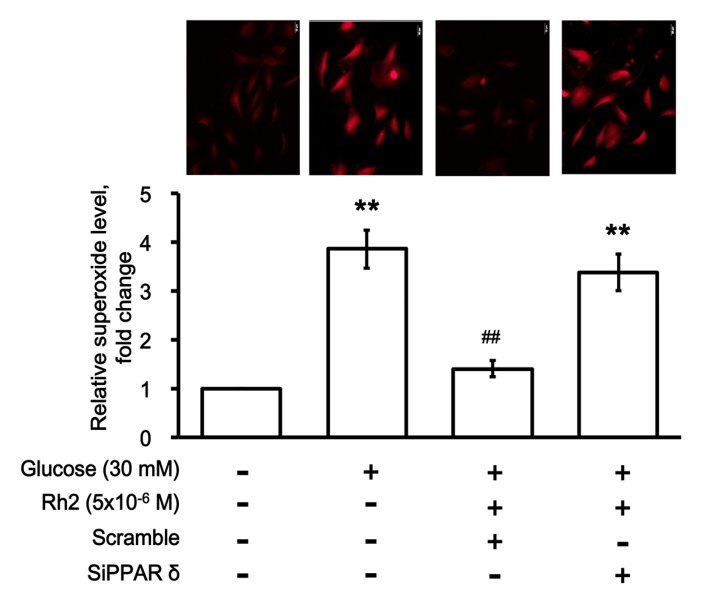
Effects of ginsenoside Rh2 on the superoxide levels in cultured H9c2 cells. The accumulation of superoxides in H9c2 cells was detected via dihydroergotamine (DHE) probes. Representative images (upper) and quantification of the relative superoxide levels (lower) in H9c2 cells calculated based on the fluorescence intensity are indicated. The fluorescence images were analyzed with the NIH ImageJ software. Changes in H9c2 cells transfected with siRNA specific for PPARδ (SiPPARδ) were compared with those receiving blank vector only (Scramble). The results are presented as the mean ± SEM (*n* = 6 per group). ** *p* < 0.01 compared with the control (first column) and ^##^ *p* < 0.01 compared with the vehicle-treated cells under high glucose media (second column) (two-way ANOVA with a post hoc Tukey test). Scar bar: 50 µm.

The effect of Rh2 on oxidative damage was investigated via the detection of the intracellular superoxide ion (ROS) levels. Following its introduction into cells, the superoxide anion reacts with dihydroergotamine (DHE), which generates a red fluorescent product 2-hydroxyethidium (EOH). As shown in Figure 6, incubation in high glucose (30 mM) media increased the generation of superoxide ions in the H9c2 cells as determined using the calculation of fluorescence intensity. Rh2 attenuated the effects of high glucose on the generation of superoxide ions. However, as shown in Figure 6, the effectiveness of Rh2 disappeared in the PPARδ gene-silenced H9c2 cells.

## 3. Discussion

In the present study, for the first time, we demonstrated that ginsenoside Rh2 may alleviate cardiac fibrosis induced by hyperglycemia both in vivo and in vitro and identified that the effect of Rh2 is mainly induced via mediation of the PPARδ signaling pathway.

The expression levels of p-STAT3/STAT3, CCN2 and fibronectin in heart tissues from STZ-diabetic rats significantly increased. Similar increases in the expression levels of these fibrotic signals were also characterized in a cardiac cell line treated with high glucose media. Therefore, high glucose may promote the expression of CCN2 and fibronectin following the activation of STAT3 (p-STAT3/STAT3). Moreover, Rh2 diminished high glucose-induced CCN2 and fibronectin expression and STAT3 activation. Cardiac performances (+dP/dt_max_; −dP/dt_max_) were also substantially improved by Rh2 in STZ-diabetic rats. These effects of Rh2 were reversed by GSK0660 at an effective dose to inhibit PPARδ. The mediation of PPARδ signaling in Rh2-induced changes in cardiac function was further supported by the silencing of PPARδ with siRNA in addition to the direct elevation of PPARδ expression levels by Rh2. Therefore, we report a novel view that Rh2 has the ability to activate PPARδ and thus, may attenuate the fibrosis-related gene expression in diabetic rats.

Diabetic complications are mainly related to hyperglycemia [25]. In the present study, the plasma glucose level was substantially higher in the STZ-diabetic rats than in the normal rats, indicating the success of diabetes model induction. The cardiac weight index in the STZ-diabetic rats was significantly increased, indicating the presence of myocardial hypertrophy. Interstitial fibrosis is a key pathological change that contributes to the impairment of cardiac function during diabetic cardiomyopathy [26]. Increased gene expression of collagen I and collagen III was identified in the hearts of the STZ rats, which were reduced after Rh2 administration. Furthermore, histological data indicated irregularly arranged, hypertrophic and twisted myocardial cells, as well as widened intercellular spaces and cardiac fibrosis in the STZ-diabetic rats. Moreover, Masson’s staining showed less fibrotic deposition in the hearts of the Rh2-treated STZ-induced diabetic rats than that in the hearts of vehicle-treated rats. Therefore, Rh2 alleviates cardiac fibrosis in diabetic rats likely as a result of the attenuation of hyperglycemia as described in a previous report [19]. However, these effects of Rh2 were reversed by the blockade of PPARδ indicating the potential role of PPARδ signaling in the effects of Rh2.

We subsequently focused on the fibrotic signals in diabetic rats and cultured cardiomyocytes. Cardiac fibroblasts within the connect tissue regulate the homeostasis of the extracellular matrix (ECM). After an injury, such as hyperglycemia, they may change to the active form to participate in cardiac fibrosis [27]. The ECM protein family includes collagens, fibronectin, and CCN2. CCN2 is established as the mediator of TGF-β during the fibrotic response [28]. Similar to collagens, fibronectin is also stimulated by high glucose levels in cardiac fibroblasts [29]. Therefore, following a previous report [30], we focused on the changes of CCN2 and fibronectin as fibrotic signals in the present study. High glucose-induced CCN2 and fibronectin expression has been characterized in vitro [30,31]. In addition to improving cardiac fibrosis, Rh2 reversed the higher CCN2 and fibronectin expression levels in the STZ-diabetic rats. Similar results were obtained in the high glucose-treated cardiac cells. Therefore, Rh2 is useful in reducing cardiac fibrosis induced by hyperglycemia. This finding is consistent with the effect of cryptotanshinone, which alleviated angiotensin II-induced cardiac fibrosis [32]. Interestingly, the effect of Rh2 appears to be associated with PPARδ because the blockade of PPARδ inhibited the effectiveness of Rh2.

STAT3 belongs to the STAT family, which includes transcription factors that act in the cytoplasm and function as major mediators of growth factors together with cytokines [33]. In cardiac functions, STAT3 exhibits contrasting effects (protective and pro-fibrotic); however, STAT3 is important in the cell-to-cell communication of the heart [34]. Moreover, STAT3 is involved in the regulation of gene synthesis for ECM homeostasis, such as collagen and various cytokines, in cardiac fibroblasts. Therefore, activated STAT3 has been shown to increase the expression of CCN2 [35] and fibronectin [36]. In the present study, increased expression levels of CCN2 and fibronectin and STAT3 activation (p-STAT3/STAT3) were identified in the heart tissues of STZ-treated rats. Furthermore, the expression levels of CCN2 and fibronectin in addition to STAT3 activation were also enhanced by high glucose treatment in cultured cardiac cells. Therefore, we demonstrated that STAT3 activation and expression of CCN2 and fibronectin are enhanced by hyperglycemia in the heart both in vivo and in vitro. Moreover, Rh2 reduced these changes in the heart in a PPARδ-dependent manner.

Peroxisome proliferator-activated receptors, PPARs (α, δ and γ), belong to the nuclear receptor family of transcription factors. Compared with other PPARs, PPARδ exhibits higher expression in the heart [37]. Cardiac PPARδ deletion induces cardiac dysfunction, hypertrophy, and heart failure in rodents [38]. Activation of PPARδ by an agonist may improve phenylephrine-induced hypertrophy in cardiomyocytes [39]. Furthermore, ginseng has been demonstrated to improve heart function via PPARδ in diabetic rats [21]. In the present study, PPARδ is substantially reduced in the hearts of diabetic rats. Moreover, we show that Rh2 reversed the lower PPARδ expression in the heart both in vivo and in vitro. In the present study, Rh2 is effective in ameliorating cardiac fibrosis and decreasing STAT3 activation and the expression of fibrotic signals, CCN2 and fibronectin through increased PPARδ expression. This is similar to the effect of telmisartan, as described in our recent report [12]. The activation of PPARδ has been demonstrated to inhibit cardiac fibroblast proliferation and transformation to myofibroblasts [40]. The activation of PPARδ has also been proposed as a potential treatment of cardiac fibrosis [41]. Both of these reports are consistent with our findings. However, the details regarding the activation of PPARδ induced by Rh2 will be investigated in the future.

An impaired left ventricular ejection fraction (LVEF) and enhanced LV remodeling, inflammation, and fibrosis have been widely observed in diabetic animals [20]. The changed cardiac performance was substantially improved by Rh2, which was also inhibited by the pharmacological blockade of PPARδ in STZ-diabetic rats. Mediation of PPARδ in Rh2-induced cardiac contraction may thus be identified in diabetic rats. This is similar to our previous finding [42] that the activation of PPARδ enhanced cardiac contractility. However, the activation of PPARδ by Ramipril failed to restore cardiac function in anthracycline-induced cardiomyopathy [43]. Therefore, the effectiveness of an agonist to activate PPARδ against cardiac damage appears different and will be characterized in the future.

In diabetic rats, cardiac apoptosis has been documented to associate with the loss of cardiomyocytes and it was generally initiated at the early stage after injection of streptozotocin (STZ) [44]. STZ-diabetic rats showed a significant increase in the levels of nitric oxide (NO), compared with the normal rats [45]. NO in known to interact with ROS to form reactive nitrogen species, which may further damage the mitochondrial function and can lead to open the mitochondrial permeability transition pore to result in cell death [46]. Rh2 as an effective agent displayed the protective effect against the cytotoxicity in vitro and in vivo [19]. Decreased cardiac PPARδ expression has been identified in rats that exhibit diabetic cardiomyopathy [47], and the reduction of PPARδ expression in cardiac cells during hyperglycemia has been found to be associated with higher reactive oxygen species (ROS) production [42]. Furthermore, hyperglycemia may directly increase cardiac fibroblast proliferation [29]. Therefore, ROS induced by hyperglycemia appear important in diabetic cardiomyopathy as previously described [48]. In the present study, the effect of Rh2 on the superoxide level was also investigated in H9c2 cells treated with high glucose media. Similar to a previous report [49], a marked production of superoxide levels was identified in H9c2 cells incubated with high glucose media. Treatment with Rh2 as well as PPARδ blockade using siRNA attenuated the formation of superoxide levels. Reduction of superoxide or ROS by Rh2 via PPARδ may thus be considered. The merits of PPARδ activation by agonists have been suggested in rat models of myocardial ischemia/reperfusion injury [50], in addition to the reduction of oxidative stress-induced apoptosis in H9c2 cells [51]. A major role for PPARδ in the prevention of oxidative stress in the brain has also been identified using knockout mice [52]. Therefore, Rh2 activating PPARδ to inhibit oxidative stress may be considered one of the main mechanisms in the reduction of cardiac fibrosis in diabetic rats.

Moreover, administration of Rh2 at a dose (120 mg/kg) exhibited a peak plasma concentration of Rh2 approximately 19.0 ± 2.0 μg/mL in nude mice [53]. The bioavailability of Rh2 is introduced to about 5% in rats and 16% in dogs [54]. However, Rh2 belonged to a small proportion of the content in ginseng herb. Therefore, the rationale for the dose of ginsenoside Rh2 administered in clinics and the relationship with dietary intake still need the additional studies in advance. In the present study, the two series of experiments were quite different, mainly in the time of exposure to both high glucose and Rh2. It is important to define the effective dose of Rh2 in daily intake.

In conclusion, increased CCN2 and fibronectin expression levels in parallel with STAT3 activation are identified in diabetic cardiomyopathy. Moreover, we demonstrated a novel finding that Rh2 may activate PPARδ to inhibit these fibrotic signals to ameliorate the impaired heart function and cardiac fibrosis induced by hyperglycemia.

## 4. Materials and Methods

### 4.1. Animals and Ethical Statement

All animal care and procedures for the in vivo studies complied with the UK Home Office Guidelines on the Animals (Scientific Procedures) Act 1986 (published by the Stationery Office, London, UK); the project license (Protocol 105122615, 26 December 2016) was approved by the Institutional Animal Ethics Committee of the Chi-Mei Medical Center. Male Sprague-Dawley (SD) rats that weighed 250 to 270 g were obtained from the National Laboratory Animal Center (Taipei, Taiwan). In addition to the experiments performed under anesthesia with sodium pentobarbital (35 mg/kg) via intraperitoneal injection (i.p.), all efforts were made to minimize animal suffering. All experiments conformed with the Guide for the Care and Use of Laboratory Animals, as well as the guidelines of the Animal Welfare Act.

### 4.2. Diabetic Rat Model

Diabetes was induced via an intravenous (i.v.) injection of streptozotocin (65 mg/kg) into fasting rats as previously described [22]. Animals were considered diabetic once they exhibited a plasma glucose level greater than 320 mg/dL. All studies were performed 8 weeks after diabetes induction.

### 4.3. Treatment Protocols

The rats were separated into four groups (*n* = 8): normal rats (control); vehicle-treated STZ-diabetic rats (STZ); Rh2-treated STZ-diabetic rats (Rh2); and GSK0660-pretreated Rh2-treated STZ-diabetic rats. In accordance with a previous study [19], Rh2 was prepared in 1% carboxymethyl cellulose sodium (CMCS) and 0.9% sodium chloride. The prepared solution or the vehicle solution was then intravenously injected at 5 mg/kg Rh2 daily for 28 days, as described in a previous report [19]. Moreover, the last group received GSK0660 that was dissolved in the same manner at 1 mg/kg 30 min prior to Rh2 administration. Cardiac performance was measured, and the hearts were harvested at the end of the study.

### 4.4. Materials

Ginsenoside Rh2 was obtained from Tauto Biotech (Shanghai, China). The purity was greater than 98% as determined by high performance liquid chromatography (HPLC), according to the documentation from the manufacturer.

### 4.5. Hemodynamic Measurements

Hemodynamic measurements were performed following a previous report [55]. Briefly, temporary pacing leads were placed in the right atrium and the RV apex of the heart. An LV pacing electrode (IX-214; iWorx Systems, Inc., Dover, NH, USA) was placed in the anterior region via the great cardiac vein. Pressure transducer catheters were subsequently inserted into the heart. Ventricular pressure signals were recorded with a data acquisition system (iWorx Systems, Inc., Dover, NH, USA). The hemodynamic indices were calculated as the mean of all beats during a 20-s steady-state period. The following indices were obtained: heart rate (HR), LV systolic pressure (LVSP), LV end-diastolic pressure (LVEDP), and maximal and minimal rates of LV pressure change (+dp/dt_max_ and −dp/dt_max_, respectively). All rats were maintained at 37.5 °C during the experiments. The rats were sacrificed after the analysis of myocardial function. The hearts were harvested for the subsequent studies.

### 4.6. Determination of Cardiac Weight Index

The cardiac weight index was predominately determined by following a previous study [56]. The whole heart was isolated from rats under anesthesia to rinse with saline solution. The left ventricle was subsequently removed and weighed (LVW). The cardiac weight index was calculated as the LVW/body weight (BW).

### 4.7. Histology of the Myocardial Tissues

Following a previous report [57], the isolated heart tissue was prepared via regular processing to embed in paraffin and cut into 5 μm-thick serial sections. One rat heart section was randomly selected from each of the 3 groups. These sections were stained with hematoxylin–eosin (HE) or Masson’s trichrome (Masson). In Masson’s trichrome staining, the collagen fibers were stained blue, and cardiomyocytes were stained red. The sections were imaged at 200× magnification by optical microscopy connected to a video camera. The myocardial collagen volume deposition was quantified using Image-Pro analysis software (Media Cybernetics, Rockville, MD, USA). In each rat, five fields of each sample obtained under a microscope were randomly selected. The averages of interstitial and perivascular fibrosis were then calculated from each group of eight rats.

### 4.8. Cell Culture and Treatment

The H9c2 cells (BCRC No. 60096) were cultured following a previous method [58]. In brief, H9c2 cells were maintained in Dulbecco’s Modified Eagle’s Medium (DMEM, pH 7.2; GIBCO-BRL Life Technologies, Gaithersburg, MD, USA) supplemented with 10% fetal bovine serum. The H9c2 cells were plated at a density of 6000 cells/cm^2^ and allowed to proliferate in the growth medium. After plating, the medium was replaced on the second day. On the next day, the cells were incubated under hyperglycemic conditions as subsequently described.

Hyperglycemia-treated cardiomyocytes were prepared by incubating the cells with 30 mM glucose for 24 h according to our previous study [23]. In brief, when the cells reached approximately 60% confluence in the culture medium supplemented with 10% fetal bovine serum, they were removed for incubation in serum-free medium that contained d-glucose (30 mM) for 24 h [23]. H9c2 cells exposed to 5.5 mM d-glucose in the same manner were used as the control. The cells were subsequently washed twice with PBS and removed from the culture dishes via trypsinization. The cells were collected from each group for analysis via Western blot.

### 4.9. Small Interfering RNA (SiRNA)

Following a previous report [12], PPARδ-specific small interference RNA (SiPPARδ) (ON-TARGETplus Rat Ppard (25682) siRNA-SMARTpool, GE Healthcare Dharmacon, Inc., Lafayette, CO, USA) or scrambled siRNA (Sc) were transfected to H9c2 cells. Transfection of the siRNA was performed using a Lipofectamine 2000 (Thermo Fisher Scientific, Pittsburgh, PA, USA). The transfected cells were incubated for 48 h prior to use. Successful transfection was confirmed using Western blots.

### 4.10. Western Blot Analysis

We used ice-cold radio-immuno-precipitation assay (RIPA) buffer to extract the proteins from rat heart homogenates or cell lysates. Western blot analysis was subsequently performed according to our previous method [59]. The target antigens from the protein extracts were detected using primary antibodies specific for PPARδ (Thermo-Fisher Sci., Rockford, IL, USA), p-STAT3, STAT3, CCN2, fibronectin (ED-A) or β-actin (Abcam, Cambridge, MA, USA). The bound primary antibodies were subsequently hybridized to horseradish peroxidase-conjugated goat anti-rabbit or anti-mouse IgGs (Calbiochem, San Diego, CA, USA), and the immunoreactive bands were developed with a chemiluminescence kit (Perkin Elmer, Waltham, MA, USA). The optical densities of the bands for PPARδ (50 kDa), p-STAT3 (88 kDa), STAT3 (88 kDa), CCN2 (38 kDa), fibronectin (270 kDa), and β-actin (43 kDa) were quantified as described in our previous report [59].

### 4.11. Real-Time Reverse-Transcription-Polymerase Chain Reaction

Similar to our previous method [59], the mRNA expression levels of each signal were determined. In brief, total RNA was extracted from the cell lysates with TRIzol reagent (Carlsbad, CA, USA). Total RNA (200 ng) was reverse-transcribed into cDNA with random hexamer primers (Roche Diagnostics, Mannheim, Germany). All PCR experiments were performed using a LightCycler (Roche Diagnostics GmbH, Mannheim, Germany). The concentration of each PCR product was calculated relative to a corresponding standard curve. The relative gene expression was subsequently indicated as the ratio of the target gene level to that of β-actin. The primers for collagen type I, collagen type III, and β-actin are listed as follows:

collagen I (α1) F: 5′-CTGGCAAGAACGGAGATGAT-3’;
collagen I (α1) R: 5′-AATCCACGAGCACCCTGA-3′;
collagen III (α1) F: 5′-CAGGAAACCCAGGAGAAAGG-3′;
collagen III (α1) R: 5′-CCAGCTACTCCTATAGGTCCTG-3′;
PPARδ R: 5′-TTCCATGACTGACCCCACTT-3′;
PPARδ L: 5′-GAGGACAAACCCACGGTAAA-3′;
β-actin F: 5′-CTAAGGCCAACCGTGAAAAG-3′;
β-actin R: 5′-GCCTGGATGGCTACGTACA-3′.

### 4.12. Identification of Intracellular Superoxide Levels

Following the methods described in a previous report [60], H9c2 cells were seeded in 24-well plates at a density of 7.5 × 10^3^ cells/mL overnight. After incubation in high glucose (30 mM) media for 24 h, the cells were treated with Rh2 at the indicated concentration for an additional 24 h. For the detection of the intracellular superoxide levels, we applied DHE from Thermo Fisher Scientific Inc. (Rockford, IL, USA) to react with intracellular superoxide ions at 37 °C for 30 min. An entire field of vision was characterized using a fluorescence microscope connected to an imaging system (IX71 Olympus, Tokyo, Japan). The results were subsequently expressed as a percentage of the intracellular superoxide level in the cells based on the analysis using the NIH ImageJ software (Available online: http://imagej.nih.gov/ij/).

### 4.13. Statistical Analysis

The results are presented as the means ± SEM from the indicated sample size (*n*) in each group. Statistical analysis was performed using two-way analysis of variance (ANOVA), followed by Tukey’s post-hoc analysis to compare the differences among the diabetic/non-diabetic (pathology) and treatment (untreated/Rh2-treated) groups. *p* < 0.05 was considered significant.

## Figures and Tables

**Table 1:** Effects of ginsenoside Rh2 on the changes in blood glucose levels, body weight, heart weight, cardiac weight index, and cardiac performance.

Group	Control		STZ Rats		
	+Vehicle	+Rh2	+Vehicle	+Rh2	+Rh2 +GSK0660
Blood glucose (mg/dL)	101.4 ± 4.0	99.3 ± 5.3	328.1 ± 13.0 **	237.4 ± 15.9 **^,#^	315.3 ± 14.8 **
Body weight (g)	363.4 ± 6.5	348.9 ± 7.3	271.1 ± 6.6 **	304.0 ± 6.5 *^,#^	275.8 ± 8.2 **
Heart weight (g)	1.28 ± 0.04	1.17 ± 0.07	1.05 ± 0.04 *	0.86 ± 0.04 **^,#^	1.08 ± 0.06 **
Cardiac weight index (mg/g)	3.52 ± 0.14	3.35 ± 0.16	3.89 ± 0.18 *	2.85 ± 0.16 *^,#^	3.91 ± 0.22 *
Heart rate (beats /min)	379.50 ± 21.46	384.25 ± 26.87	289.25 ± 31.95 *	330.38 ± 24.73 *^,#^	294.50 ± 22.49 *
LVSP (mmHg)	144.4 ± 4.6	145.7 ± 5.2	113.6 ± 3.0 *	139.5 ± 3.5 *^,#^	112.1 ± 4.0 *
LVEDP (mmHg)	4.77 ± 0.46	4.95.1 ± 0.31	8.94 ± 0.32 *	5.11 ± 0.45 *^,#^	8.82 ± 0.56 *
**Cardiac Performance**					
+dp/dtmax (mmHg/S)	2525.9 ± 92.4	2602.6 ± 61.2	2089.4 ± 58.4 **	2301.1 ± 53.1 *^,#^	2106.4 ± 46.6 **
−dp/dtmax (mmHg/S)	1501.1 ± 61.5	1458.8 ± 46.3	931.3 ± 51.3 **	1246.7 ± 35.5 *^,#^	967.3 ± 69.5 **

Values are expressed as mean ± SEM (*n* = 8). The vehicle used to dissolve the tested drugs was administered at the same volume to streptozotocin-induced type-1 diabetic rats (STZ-diabetic rats). * *p* < 0.05 and ** *p* < 0.01 compared with the values obtained from the normal control group. ^#^ *p* < 0.05 compared with the values obtained from the vehicle-treated STZ-induced diabetic rats (STZ rats + Vehicle) (two-way ANOVA with a post hoc Tukey test). Left ventricular systolic pressure (LVSP); left ventricular end-diastolic pressure (LVEDP); maximum rate of left ventricle pressure rise (+dP/dt max); maximum rate of left ventricle pressure fall (−dP/dt max).

## Abbreviations

CCN2	Connective tissue growth factor
DCM	Diabetic cardiomyopathy
DHE	Dihydroergotamine
ECM	Extracellular matrix
HW/BW	Ratio, heart weight and body weight ratio
STAT3	Signal transducer and activator of transcription 3
STZ	Streptozotocin

## References

[1] Bauters C., Lamblin N., Mc Fadden E.P., Van Belle E., Millaire A., de Groote P. (2003). Influence of diabetes mellitus on heart failure risk and outcome. Cardiovasc. Diabetol..

[2] Solang L., Malmberg K., Ryden L. (1999). Diabetes mellitus and congestive heart failure. Further knowledge needed. Eur. Heart J..

[3] Huynh K., McMullen J.R., Julius T.L., Tan J.W., Love J.E., Cemerlang N., Kiriazis H., Du X.J., Ritchie R.H. (2010). Cardiac-specific IGF-1 receptor transgenic expression protects against cardiac fibrosis and diastolic dysfunction in a mouse model of diabetic cardiomyopathy. Diabetes.

[4] Falcao-Pires I., Leite-Moreira A.F. (2012). Diabetic cardiomyopathy: Understanding the molecular and cellular basis to progress in diagnosis and treatment. Heart Fail. Rev..

[5] Brownlee M. (2005). The pathobiology of diabetic complications: A unifying mechanism. Diabetes.

[6] Mair M., Blaas L., Osterreicher C.H., Casanova E., Eferl R. (2011). Jak-stat signaling in hepatic fibrosis. Front. Biosci..

[7] Radhakrishnan S.S., Blalock T.D., Robinson P.M., Secker G., Daniels J., Grotendorst G.R., Schultz G.S. (2012). Effect of connective tissue growth factor on protein kinase expression and activity in human corneal fibroblasts. Investig. Ophthalmol. Vis. Sci..

[8] Wang L., Yuan T., Du G., Zhao Q., Ma L., Zhu J. (2014). The impact of 1,25-dihydroxyvitamin D3 on the expression of connective tissue growth factor and transforming growth factor-β1 in the myocardium of rats with diabetes. Diabetes Res. Clin. Pract..

[9] White E.S., Baralle F.E., Muro A.F. (2008). New insights into form and function of fibronectin splice variants. J. Pathol..

[10] Liu Y., Liu H., Meyer C., Li J., Nadalin S., Konigsrainer A., Weng H., Dooley S., ten Dijke P. (2013). Transforming growth factor-β (TGF-β)-mediated connective tissue growth factor (CTGF) expression in hepatic stellate cells requires Stat3 signaling activation. J. Biol. Chem..

[11] O’Sullivan K.E., Breen E.P., Gallagher H.C., Buggy D.J., Hurley J.P. (2016). Understanding Stat3 signaling in cardiac ischemia. Basic Res. Cardiol..

[12] Chang W.T., Cheng J.T., Chen Z.C. (2016). Telmisartan improves cardiac fibrosis in diabetes through peroxisome proliferator activated receptor δ (PPARδ): From bedside to bench. Cardiovasc. Diabetol..

[13] Lee C.H., Kim J.H. (2014). A review on the medicinal potentials of ginseng and ginsenosides on cardiovascular diseases. J. Ginseng Res..

[14] Ku S. (2016). Finding and producing probiotic glycosylases for the biocatalysis of ginsenosides: A mini review. Molecules.

[15] Sun Y., Liu Y., Chen K. (2016). Roles and mechanisms of ginsenoside in cardiovascular diseases: Progress and perspectives. Sci. China Life Sci..

[16] Park E.K., Choo M.K., Oh J.K., Ryu J.H., Kim D.H. (2004). Ginsenoside Rh2 reduces ischemic brain injury in rats. Biol. Pharm. Bull..

[17] Kim J.H., Choi J.S. (2016). Effect of ginsenoside Rh-2 via activation of caspase-3 and Bcl-2-insensitive pathway in ovarian cancer cells. Physiol. Res..

[18] Li B., Zhao J., Wang C.Z., Searle J., He T.C., Yuan C.S., Du W. (2011). Ginsenoside rh2 induces apoptosis and paraptosis-like cell death in colorectal cancer cells through activation of p53. Cancer Lett..

[19] Wang H., Yu P., Gou H., Zhang J., Zhu M., Wang Z.H., Tian J.W., Jiang Y.T., Fu F.H. (2012). Cardioprotective effects of 20(s)-ginsenoside Rh2 against doxorubicin-induced cardiotoxicity in vitro and in vivo. Evid. Based Complement. Altern. Med..

[20] Wang H.T., Liu C.F., Tsai T.H., Chen Y.L., Chang H.W., Tsai C.Y., Leu S., Zhen Y.Y., Chai H.T., Chung S.Y. (2012). Effect of obesity reduction on preservation of heart function and attenuation of left ventricular remodeling, oxidative stress and inflammation in obese mice. J. Trans. Med..

[21] Tsai C.C., Chan P., Chen L.J., Chang C.K., Liu Z., Lin J.W. (2014). Merit of ginseng in the treatment of heart failure in type 1-like diabetic rats. BioMed Res. Int..

[22] Cheng J.T., Huang C.C., Liu I.M., Tzeng T.F., Chang C.J. (2006). Novel mechanism for plasma glucose-lowering action of metformin in streptozotocin-induced diabetic rats. Diabetes.

[23] Chen Z.C., Yu B.C., Chen L.J., Cheng K.C., Lin H.J., Cheng J.T. (2011). Characterization of the mechanisms of the increase in PPARδ expression induced by digoxin in the heart using the H9c2 cell line. Br. J. Pharmacol..

[24] Cai J., Chen X., Chen X., Chen L., Zheng G., Zhou H., Zhou X. (2017). Anti-fibrosis effect of relaxin and spironolactone combined on isoprenaline-induced myocardial fibrosis in rats via inhibition of endothelial-mesenchymal transition. Cell. Physiol. Biochem..

[25] Wang C.M., Hsu C.T., Niu H.S., Chang C.H., Cheng J.T., Shieh J.M. (2016). Lung damage induced by hyperglycemia in diabetic rats: The role of signal transducer and activator of transcription 3 (Stat3). J. Diabetes Complicat..

[26] Goyal B.R., Mehta A.A. (2013). Diabetic cardiomyopathy: Pathophysiological mechanisms and cardiac dysfuntion. Hum. Exp. Toxicol..

[27] Travers J.G., Kamal F.A., Robbins J., Yutzey K.E., Blaxall B.C. (2016). Cardiac fibrosis: The fibroblast awakens. Circ. Res..

[28] Souders C.A., Bowers S.L., Baudino T.A. (2009). Cardiac fibroblast: The renaissance cell. Circ. Res..

[29] Tokudome T., Horio T., Yoshihara F., Suga S., Kawano Y., Kohno M., Kangawa K. (2004). Direct effects of high glucose and insulin on protein synthesis in cultured cardiac myocytes and DNA and collagen synthesis in cardiac fibroblasts. Metab. Clin. Exp..

[30] Liu Y., Qi H., Wang Y., Wu M., Cao Y., Huang W., Li L., Ji Z., Sun H. (2012). Allicin protects against myocardial apoptosis and fibrosis in streptozotocin-induced diabetic rats. Phytomedicine Int. J. Phytother. Phytopharmacol..

[31] Chavali V., Nandi S.S., Singh S.R., Mishra P.K. (2014). Generating double knockout mice to model genetic intervention for diabetic cardiomyopathy in humans. Methods Mol. Biol..

[32] Ma Y., Li H., Yue Z., Guo J., Xu S., Xu J., Jia Y., Yu N., Zhang B., Liu S. (2014). Cryptotanshinone attenuates cardiac fibrosis via downregulation of Cox-2, Nox-2, and Nox-4. J. Cardiovasc. Pharmacol..

[33] Artas G., Ozercan H.I. (2014). The expression of stat3, Bcl-Xl and Mmp-2 proteins in colon adenocarcinomas and their relationship with prognostic factors. Turk. Patoloji Dergisi.

[34] Haghikia A., Ricke-Hoch M., Stapel B., Gorst I., Hilfiker-Kleiner D. (2014). Stat3, a key regulator of cell-to-cell communication in the heart. Cardiovasc. Res..

[35] Du J., Wang L., Liu X., Zhou H., Fan Q., Luo J., Yao L., Wang J., Feng J., Ma J. (2010). Janus kinase 2/signal transducers and activators of transcription signal inhibition regulates protective effects of probucol on mesangial cells treated with high glucose. Biol. Pharm. Bull..

[36] Tang M., Zhang W., Lin H., Jiang H., Dai H., Zhang Y. (2007). High glucose promotes the production of collagen types i and iii by cardiac fibroblasts through a pathway dependent on extracellular-signal-regulated kinase 1/2. Mol. Cell. Biochem..

[37] Gilde A.J., van der Lee K.A., Willemsen P.H., Chinetti G., van der Leij F.R., van der Vusse G.J., Staels B., van Bilsen M. (2003). Peroxisome proliferator-activated receptor (PPAR) α and PPARβ/δ, but not PPARγ, modulate the expression of genes involved in cardiac lipid metabolism. Circ. Res..

[38] Cheng L., Ding G., Qin Q., Huang Y., Lewis W., He N., Evans R.M., Schneider M.D., Brako F.A., Xiao Y. (2004). Cardiomyocyte-restricted peroxisome proliferator-activated receptor-δ deletion perturbs myocardial fatty acid oxidation and leads to cardiomyopathy. Nat. Med..

[39] Planavila A., Rodriguez-Calvo R., Jove M., Michalik L., Wahli W., Laguna J.C., Vazquez-Carrera M. (2005). Peroxisome proliferator-activated receptor β/δ activation inhibits hypertrophy in neonatal rat cardiomyocytes. Cardiovasc. Res..

[40] Teunissen B.E., Smeets P.J., Willemsen P.H., De Windt L.J., Van der Vusse G.J., Van Bilsen M. (2007). Activation of PPARδ inhibits cardiac fibroblast proliferation and the transdifferentiation into myofibroblasts. Cardiovasc. Res..

[41] Wagner K.D., Wagner N. (2010). Peroxisome proliferator-activated receptor β/δ (PPARβ/δ) acts as regulator of metabolism linked to multiple cellular functions. Pharmacol. Ther..

[42] Chen Z.C., Lee K.S., Chen L.J., Wang L.Y., Niu H.S., Cheng J.T. (2013). Cardiac peroxisome proliferator-activated receptor δ (PPARδ) as a new target for increased contractility without altering heart rate. PLoS ONE.

[43] Cernecka H., Doka G., Srankova J., Pivackova L., Malikova E., Galkova K., Kyselovic J., Krenek P., Klimas J. (2016). Ramipril restores PPARβ/δ and PPARγ expressions and reduces cardiac nadph oxidase but fails to restore cardiac function and accompanied myosin heavy chain ratio shift in severe anthracycline-induced cardiomyopathy in rat. Eur. J. Pharmacol..

[44] Ghosh S., Ting S., Lau H., Pulinilkunnil T., An D., Qi D., Abrahani M.A., Rodrigues B. (2004). Increased efflux of glutathione conjugate in acutely diabetic cardiomyocytes. Can. J. Physiol. Pharmacol..

[45] Napoli C., Ignarro L.J. (2009). Nitric oxide and pathogenic mechanisms involved in the development of vascular diseases. Arch. Pharm. Res..

[46] Amin K.A., Awad E.M., Nagy M.A. (2011). Effects of panax quinquefolium on streptozotocin-induced diabetic rats: Role of c-peptide, nitric oxide and oxidative stress. Int. J. Clin. Exp. Med..

[47] Lee T.I., Kao Y.H., Chen Y.C., Pan N.H., Chen Y.J. (2010). Oxidative stress and inflammation modulate peroxisome proliferator-activated receptors with regional discrepancy in diabetic heart. Eur. J. Clin. Investig..

[48] Huynh K., Bernardo B.C., McMullen J.R., Ritchie R.H. (2014). Diabetic cardiomyopathy: Mechanisms and new treatment strategies targeting antioxidant signaling pathways. Pharmacol. Ther..

[49] Chen J., Guo R., Yan H., Tian L., You Q., Li S., Huang R., Wu K. (2014). Naringin inhibits Ros-activated mapk pathway in high glucose-induced injuries in H9c2 cardiac cells. Basic Clin. Pharmacol. Toxicol..

[50] Kapoor A., Collino M., Castiglia S., Fantozzi R., Thiemermann C. (2010). Activation of peroxisome proliferator-activated receptor-β/δ attenuates myocardial ischemia/reperfusion injury in the rat. Shock.

[51] Pesant M., Sueur S., Dutartre P., Tallandier M., Grimaldi P.A., Rochette L., Connat J.L. (2006). Peroxisome proliferator-activated receptor δ (PPARδ) activation protects H9c2 cardiomyoblasts from oxidative stress-induced apoptosis. Cardiovasc. Res..

[52] Kocalis H.E., Turney M.K., Printz R.L., Laryea G.N., Muglia L.J., Davies S.S., Stanwood G.D., McGuinness O.P., Niswender K.D. (2012). Neuron-specific deletion of peroxisome proliferator-activated receptor δ (PPARδ) in mice leads to increased susceptibility to diet-induced obesity. PLoS ONE.

[53] Musende A.G., Eberding A., Wood C., Adomat H., Fazli L., Hurtado-Coll A., Jia W., Bally M.B., Guns E.T. (2009). Pre-clinical evaluation of Rh2 in PC-3 human xenograft model for prostate cancer in vivo: Formulation, pharmacokinetics, biodistribution and efficacy. Cancer Chemother Pharmacol..

[54] Gu Y., Wang G.J., Sun J.G., Jia Y.W., Wang W., Xu M.J., Lv T., Zheng Y.T., Sai Y. (2009). Pharmacokinetic characterization of ginsenoside Rh2, an anticancer nutrient from ginseng, in rats and dogs. Food Chem. Toxicol..

[55] Seldinger S.I. (2008). Catheter replacement of the needle in percutaneous arteriography. A new technique. Acta Radiol. Suppl..

[56] Tang S.Y., Peng D.F., Hu Y.J., Chen J. (2015). Protective effects of valsartan and benazepril combined with atorvastatin on cardiorenal syndrome in rats. Eur. Rev. Med. Pharmacol. Sci..

[57] Li C.J., Lv L., Li H., Yu D.M. (2012). Cardiac fibrosis and dysfunction in experimental diabetic cardiomyopathy are ameliorated by α-lipoic acid. Cardiovasc. Diabetol..

[58] Ghosh R., Hwang S.M., Cui Z., Gilda J.E., Gomes A.V. (2016). Different effects of the nonsteroidal anti-inflammatory drugs meclofenamate sodium and naproxen sodium on proteasome activity in cardiac cells. J. Mol. Cell. Cardiol..

[59] Yeh M.C., Chen L.J., Niu H.S., Yang T.T., Lin K.C., Cheng J.T. (2014). Signals for increase of mu-opioid receptor expression in muscle by hyperglycemia. Neurosci. Lett..

[60] Cheng Y., Xia Z., Han Y., Rong J. (2016). Plant natural product formononetin protects rat cardiomyocyte H9c2 cells against oxygen glucose deprivation and reoxygenation via inhibiting ROS formation and promoting GSK-3β phosphorylation. Oxidative Med. Cell. Longev..

